# Improving TB control: efficiencies of case-finding interventions in Nigeria

**DOI:** 10.5588/pha.23.0028

**Published:** 2023-09-21

**Authors:** A. P. Babayi, B. B. Odume, C. L. Ogbudebe, O. Chukwuogo, N. Nwokoye, C. C. Dim, S. Useni, D. Nongo, R. Eneogu, O. Chijioke-Akaniro, C. Anyaike

**Affiliations:** 1 KNCV Nigeria, Abuja;; 2 College of Medicine, University of Nigeria Ituku-Ozalla, Enugu, Nigeria;; 3 United States Agency for International Development Nigeria, Abuja, Nigeria;; 4 National Tuberculosis, Leprosy and Buruli Ulcer Control Programme, Abuja, Nigeria

**Keywords:** presumptive TB yield, TB interventions, KNCV Nigeria, TB case yield efficiency, portable digital X-ray, WHO 4-symptom screen

## Abstract

**SETTING:**

KNCV Nigeria implements seven key TB case-finding interventions. It was critical to evaluate the efficiency of these interventions in terms of TB yield to direct future prioritisation in the country.

**OBJECTIVES:**

To compare the efficiency of active case-finding (ACF) interventions for TB in Nigeria.

**DESIGN:**

Data from the 2020–2022 implementing period were analysed retrospectively. Intervention efficiencies were analysed using the number needed to screen (NNS), the number needed to test (NNT) and the true screen-positive (TSP) rate.

**RESULTS:**

Across the interventions, 21,704,669 persons were screened for TB, 1,834,447 (8.5%) were presumed to have TB (7.7% pre-diagnostic drop-out rate) and 122,452 were diagnosed with TB (TSP rate of 7.2%). The average TSP rate of interventions that used both the WHO four-symptom screen (W4SS) and portable digital X-ray (PDX) screening algorithm was significantly higher (22.6%) than those that employed the former alone (7.0%; OR 3.9, 95% CI 3.74–3.98; *P* < 0.001). The average NNT for interventions with W4SS/PDX screening was 4 (range: 4–5), while that of W4SS-only screening was 14 (range: 11–22).

**CONCLUSIONS:**

Interventions using the PDX in addition to W4SS for TB screening were more efficient in terms of TB case yield than interventions that used symptom-based TB screening only.

## Abstract (fr)

**CONTEXTE:**

KNCV Nigeria met en œuvre sept interventions clés de recherche de cas de TB. Il était essentiel d’évaluer l’efficacité de ces interventions en termes de rendement de la TB afin d’orienter les priorités futures dans le pays.

**OBJECTIFS:**

Comparer l’efficacité des interventions de recherche active de cas (ACF) pour la TB au Nigéria.

**MÉTHODE:**

Les données de la période de mise en œuvre 2020–2022 ont été analysées, rétrospectivement. L’efficacité des interventions a été analysée à l’aide du nombre nécessaire pour dépister (NNS), du nombre nécessaire pour tester (NNT) et du taux de vrais dépistages positifs (TSP).

**RÉSULTATS:**

Sur l’ensemble des interventions, 21 704 669 personnes ont bénéficié d’un dépistage de la TB, 1 834 447 (8,5%) ont été présumées atteintes de la TB (taux d’abandon pré-diagnostic de 7,7%) et 122 452 ont reçu un diagnostic de TB (taux de TSP de 7,2%). Le taux moyen de TSP des interventions qui utilisaient à la fois le test des quatre symptômes de l’OMS (W4SS) et l’algorithme de dépistage par radiographie numérique portable (PDX) était significativement plus élevé (22,6%) que celles qui utilisaient uniquement le premier (7,0% ; OR 3,9 ; IC 95% 3,74–3,98 ; *P* < 0,001). Le NNT moyen des interventions avec dépistage W4SS/PDX était de 4 (intervalle : 4–5), tandis que celui du dépistage W4SS seul était de 14 (intervalle : 11–22).

**CONCLUSIONS:**

Les interventions utilisant le PDX en plus du W4SS pour le dépistage de la TB ont été plus efficaces en termes de nombre de cas de TB que les interventions utilisant uniquement le dépistage de la TB basé sur les symptômes.

The burden of TB in Nigeria remains high despite the efforts of the Nigeria national TB control programme (NTP) and its partners to meet the 2030 target of the Sustainable Development Goal 3.^1^ According to the 2022 WHO Global TB Report, the incidence of TB in Nigeria was sixth worldwide and first in Africa.^2^ The situation is worse because Nigeria is listed on the three global lists of high-burden countries for TB, HIV-associated TB and multidrug-resistant/rifampicin-resistant TB (MDR-/RR-TB) for the 2021–2025 period.^2^ Despite these negative indices, Nigeria’s performance in TB control has continued to improve over the years in line with the Nigerian NTP’s priority of identifying and treating missing TB cases.^3^ For example, it is recognised that the improved TB case detection in Nigeria in 2020/2021 is the most striking in Africa, despite the negative global impact of the COVID-19 pandemic on TB case detection.^2^

It is noteworthy that Nigeria contributes about 6.3% of the global missing TB cases and ranks fifth in the world which calls for more effort in case identification.^2^ KNCV Nigeria has responded to the notification gap in several ways, including the successful piloting of TB-loop mediated isothermal amplification (TB-LAMP) for TB diagnosis in Nigeria, the adaptation of the early warning outbreak recognition system (EWORS) for improved community active TB case-finding, etc.^4^–^6^ Because underdiagnosis is the major contributor to missing TB in Nigeria,^7^ several interventions introduced include intensified case-finding (ICF) in public and private facilities, targeted community outreaches (TCOs), portable digital X-ray (PDX) driven, active community case-finding (CCF), active case-finding (ACF) among nomads, contact investigation and Wellness on Wheels (WoW) truck-driven CCF.

With a population of over 200 million, and a TB funding deficit of nearly 70%,^8^ there is a need to evaluate interventions in Nigeria and prioritise high-yielding ones. The aim of the study was to compare the efficiency of the interventions deployed by KNCV Nigeria for active case identification during the implementation of the TB local organisation network (LON) 1 and 2 projects, across 14 states.^9^

## DESIGN AND METHODS

This study is a report based on a retrospective review of data from the ACF TB interventions implemented by the USAID-funded TB LON 1 and 2 Project for the October 2020 to December 2022 implementing period. The target communities within each state were selected purposefully based on historical data suggesting a high TB burden. Seven distinct ACF interventions were identified across the project. As shown in the Figure, each of the interventions involved three key steps:

1. Step 1: Mobilisation of individuals in the selected community for TB screening.
2. Step 2: Screening of mobilised individuals using the WHO 4-symptom screen (W4SS) comprising cough, fever, weight loss (or poor weight gain) and night sweats to identify presumptive TB,^10^ or a parallel screening using W4SS and portable digital X-ray (PDX).^11^ The PDX machine is equipped with computer-aided detection for TB (CAD4TB) software.^12^ Based on the programme cut-off point, participants with a CAD4TB score of ≥0.5 or ≥0.6 were adjudged as presumptive TB depending on the version of the CAD4TB software.
3. Step 3: Evaluation for presumptive TB. This step prioritises bacteriological methods with nucleic-acid amplification test (NAAT), especially the Xpert® MTB/RIF assay (Cepheid, Sunnyvale, CA, USA), for those who could produce specimens (sputum for adults or stool for children). Presumptive TB in those who could not produce appropriate specimens were promptly referred for clinical assessment using chest X-ray (CXR) by trained radiologists. Presumptive TB in those whose specimens tested negative for the bacteriological test, further had a clinical expert review of their CXR films (i.e., using the sequential confirmatory test algorithm).^11^ CXR for clinical diagnosis was either derived from PDX during parallel TB screening or from a radiographic service linked to the project (i.e., for presumptive screened with W4SS only).

**FIGURE:**
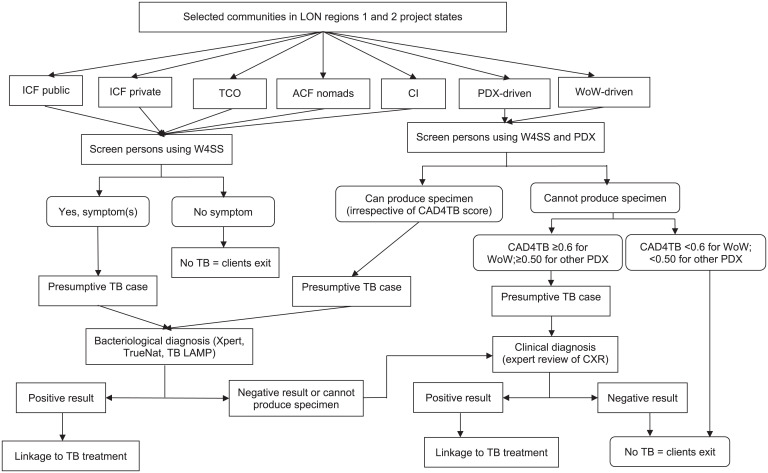
Flow diagram of community TB case-finding interventions. LON = local organisation network; ICF = intensified case-finding; TCO = targeted community outreaches; ACF = active case-finding; CI = contact investigation; PDX = portable digital X-ray; WoW = Wellness on Wheels; W4SS = WHO 4-symptom screen; CXR = chest X-ray.

As shown in the project flow diagram (Figure), all interventions differed in the methods used in Step 1 to mobilise clients for TB screening. For Step 2, respectively five vs. two interventions used the W4SS-only screening algorithm or the parallel W4SS + PDX algorithm. Step 3 (i.e., TB evaluation) was the same for all interventions. The specific TB ACF interventions compared in this report are as follows:

1. ICF among public health facilities (ICF public): this intervention involved active TB screening of clinic attendees by engaged ad hoc staff across various service delivery points at selected public healthcare facilities within the project states.
2. ICF among private health facilities (ICF private): This involved active TB screening of clinic attendees at private hospitals and individuals patronising patent medicine vendors and privately owned community pharmacies in the project states.
3. TCOs: this is also referred to as community screening and involved identifying TB hotspot areas using incidence data on a mapping software (EWORS) and conducting TB screening in targeted areas to identify presumptive TB for testing, and treatment where necessary.^6^
4. PDX-driven community ACF (PDX-driven ACF): this intervention entailed the use of artificial intelligence (AI) enabled PDX to screen individuals in the community for TB and identify presumptive for further evaluation. For this intervention, the CAD4TB score threshold for presumptive TB was ≥0.5.
5. ACF among nomads (ACF nomads): this involved identifying a specific population of nomadic herdsmen and screening them for TB. This intervention was carried out in one state only.
6. Contact investigation (CI): this investigation uses confirmed TB patients (referred to as index TB patients) as a target to identify and screen their close (household) contacts for TB.^13^
7. Wellness on Wheels (WoW) truck-driven CCF (WoW-driven CCF): this intervention used the WoW Truck to penetrate hard-to-reach communities in project states. The WoW truck is a mobile multi-diagnostic platform that combines AI-enabled CXR (for TB screening) and Xpert instrument for TB diagnosis. The CAD4TB score threshold associated with the PDX machine in the WoW truck is ≥0.6. There are two WoW trucks – one each for the northern and southern parts of Nigeria.

The outcome measure of the study was the efficiency of each TB case-finding intervention which was assessed using the number needed to screen (NNS),^14^ the number needed to test (NNT),^14^ true screen-positive (TSP) rate,^11^ and pre-diagnostic drop-out rate of presumptive TB.^15^ The NNS is the average number of individuals that need to be screened to diagnose one TB case (i.e., the ratio of persons screened to TB patients diagnosed); NNT is the average number of presumptive TB needed to be tested to identify one TB case (i.e., the ratio of presumptive evaluated to TB cases diagnosed),^14^ while pre-diagnostic drop-out of presumptive TB rate is the proportion of identified presumptive TB per intervention who did not undergo TB diagnostic evaluation, expressed as a percentage.^15^

De-identified summary data of all individuals involved in each intervention were extracted into an MS Excel (Microsoft, Redmond, WA, USA) workbook where descriptive statistics were done, while relevant inferential analyses were carried out using Open-Source Epidemiologic Statistics for Public Health (https://www.openepi.com/Menu/OE_Menu.htm). *P* < 0.05 was considered statistically significant.

### Ethical considerations

Persons recruited into each active TB case-finding intervention provided verbal consent for the clinical care. This report is a non-research programme evaluation, without direct contact with human subjects, and only de-identified pooled programme data were used.

## RESULTS

As shown in Table 1, the population of individuals screened for TB across the seven ACF interventions within the 27 months TB ACF was 21,704,669 persons (males: 44.1%, females: 55.9%). The most common age group among the screened population was 25–34 years, while the least common was ≥65 years. The ICF public intervention covered the largest population (58.2%), while the least coverage was achieved among ACF nomads (0.1%).

**TABLE 1:** Distribution of client characteristics across the ACF interventions

Intervention type	ICF public *n* (%)	ICF private *n* (%)	TCO *n* (%)	ACF nomads *n* (%)	CI *n* (%)	PDX-driven ACF *n* (%)	WoW-driven CCF *n* (%)	Sub-group total *n* (%)
Sex								
Female	7,593,976 (60.1)	3,036,092 (50.6)	1,103,960 (50.2)	10,596 (44.3)	260,707 (47.1)	58,962 (46.1)	68,500 (40.9)	12,132,793 (55.9)
Male	5,038,191 (39.9)	2,967,006 (49.4)	1,093,182 (49.8)	13,307 (55.7)	292,259 (52.9)	69,032 (53.9)	98,899 (59.1)	9,571,876 (44.1)
Age group, years								
0–4	1,764,763 (14.0)	335,884 (5.6)	137,000 (6.2)	1,442 (6.0)	36,043 (6.5)	1,115 (0.9)	909 (0.5)	2,277,156 (10.5)
5–14	1,232,658 (9.8)	652,787 (10.9)	232,621 (10.6)	2,177 (9.1)	80,232 (14.5)	12,643 (9.9)	12,546 (7.5)	2,225,664 (10.3)
15–24	1,988,643 (15.7)	933,560 (15.6)	333,373 (15.2)	2,989 (12.5)	109,518 (19.8)	22,991 (18.0)	27,176 (16.2)	3,418,250 (15.7)
25–34	2,534,115 (20.1)	1,054,006 (17.6)	383,964 (17.5)	3,394 (14.2)	108,546 (19.6)	24,921 (19.5)	36,119 (21.6)	4,145,065 (19.1)
35–44	1,946,392 (15.4)	1,002,744 (16.7)	369,115 (16.8)	3,730 (15.6)	83,694 (15.1)	23,067 (18.0)	30,831 (18.4)	3,459,573 (15.9)
45–54	1,371,381 (10.9)	835,483 (13.9)	319,700 (14.6)	3,694 (15.5)	66,013 (11.9)	18,313 (14.3)	25,755 (15.4)	2,640,339 (12.2)
55–64	1,007,291 (8.0)	674,286 (11.2)	245,019 (11.2)	3,704 (15.5)	41,001 (7.4)	13,389 (10.5)	19,550 (11.7)	2,004,240 (9.2)
≥65	786,924 (6.2)	514,348 (8.6)	176,350 (8.0)	2,773 (11.6)	27,919 (5.0)	11,555 (9.0)	14,513 (8.7)	1,534,382 (7.1)
Total	12,632,167 (58.2)	6,003,098 (27.7)	2,197,142 (10.1)	23,903 (0.1)	552,966 (2.5)	127,994 (0.6)	167,399 (0.8)	21,704,669 (1000.0)

ACF = active case-finding; ICF = intensified case-finding; TCO = targeted community outreaches; CI = contact investigation; PDX = portable digital X-ray; WoW = Wellness on Wheels; CCF = community case-finding.

Table 2 shows that 1,834,447 (8.5%) presumptive TB were identified from the population screened for TB across all the interventions. Unlike the distribution for the population screened (Table 1), the least frequent age group among those with presumptive TB was 0–4 years (0.5%). The ACF nomads yielded the highest proportion of presumptive TB per population screened (36.0%), while the least contributing intervention was WoW-driven CCF (6.4%). In all, 141,384 (7.7%) of all identified presumptive TB persons were not available for TB diagnostic testing. The pre-diagnostic drop-out rate was highest in the ICF public and TCO interventions, both at 8.2%, while the lowest dropout rate was observed in case of PDX-driven ACF. The pre-diagnostic drop-out rate was significantly higher among interventions that used W4SS only for TB screening than the two interventions (PDX-driven ACF and WoW-driven CCF) that used parallel W4SS-PDX screening (141,305/1,670,045 [8.5%] vs. 79/23,018 [0.3%]; odds ratio [OR] 24.7, 95% confidence interval [CI] 19.77–30.75; *P* < 0.001).

**TABLE 2:** Distribution of presumptive TB characteristics across the ACF interventions

**A)**								
Category	ICF public *n* (%)	ICF private *n* (%)	TCO *n* (%)	ACF nomads *n* (%)	CI *n* (%)	PDX-driven ACF *n* (%)	WoW-driven CCF *n* (%)	Sub-group total *n* (%)
Sex								
Female	448,468 (54.4)	286,665 (47.6)	117,640 (49.2)	3,549 (41.3)	68,373 (49.7)	5,229 (42.3)	3,840 (35.8)	933,764 (50.9)
Male	375,611 (45.6)	315,672 (52.4)	121,231 (50.8)	5,048 (58.7)	69,093 (50.3)	7,128 (57.7)	6,900 (64.2)	900,683 (49.1)
Age group, years								
0–4	61,936 (7.5)	12,330 (2.00)	12,432 (5.2)	281 (3.3)	8,763 (6.4)	87 (0.7)	54 (0.5)	95,883 (5.2)
5–14	80,443 (9.8)	51,096 (8.5)	28,098 (11.8)	564 (6.6)	18,686 (13.6)	858 (6.9)	498 (4.6)	180,243 (9.8)
15–24	123,161 (14.9)	107,471 (17.8)	35,380 (14.8)	1,028 (12.0)	24,333 (17.7)	1,653 (13.4)	609 (5.7)	293,635 (16.0)
25–34	158,201 (19.2)	128,621 (21.4)	42,267 (17.7)	1,354 (15.7)	25,848 (18.8)	1,998 (16.2)	1,208 (11.2)	359,497 (19.6)
35–44	141,245 (17.1)	107,594 (17.9)	38,751 (16.2)	1,529 (17.8)	21,894 (15.9)	2,070 (16.8)	1,687 (15.7)	314,770 (17.2)
45–54	109,018 (13.2)	83,628 (13.9)	33,159 (13.9)	1,494 (17.4)	16,769 (12.2)	1,708 (13.8)	1,889 (17.6)	247,665 (13.5)
55–64	80,584 (9.8)	62,497 (10.4)	26,734 (11.2)	1,405 (16.3)	12,004 (8.7)	1,611 (13.0)	2,210 (20.6)	187,045 (10.2)
≥65	69,491 (8.4)	49,100 (8.2)	22,050 (9.2)	942 (11.0)	9,169 (6.7)	2,372 (19.2)	2,585 (24.1)	1,55,709 (8.5)
Total	824,079 (44.9)	602,337 (32.8)	238,871 (13.0)	8,597 (0.5)	137,466 (7.5)	12,357 (0.7)	10,740 (0.6)	1,834,447 (100.0)
**B)** Pre-diagnostic drop-out of presumptive across ICF interventions								
	ICF public *n* (%)	ICF private *n* (%)	TCO *n* (%)	ACF nomads *n* (%)	CI *n* (%)	PDX-driven ACF *n* (%)	WoW-driven CCF *n* (%)	Sub-group total *n* (%)
Population screened, *n*	12,632,167	6,003,098	2,197,142	23,903	552,966	127,994	167,399	21,704,669
Population identified as presumptive, %	6.5	10.0	10.9	36.0	24.9	9.7	6.4	8.5
Presumptive tested for TB, *n*	756,507	554,917	219,273	8,332	131,016	12,342	10,676	1,693,063
Pre-diagnostic drop-out, *n*	67,572	47,420	19,598	265	6,450	15	64	141,384
Pre-diagnostic drop-out, %	8.2	7.9	8.2	3.1	4.7	0.1	0.6	7.7

ACF = active case-finding; ICF = intensified case-finding; TCO = targeted community outreaches; CI = contact investigation; PDX = portable digital X-ray; WoW = Wellness on Wheels; CCF = community case-finding.

Of all presumptive cases tested for TB across the seven ACF interventions, 122,452 TB cases were confirmed, giving a TSP rate of 7.2% and a case notification rate of 564/100,000 persons screened. The highest TSP rate was achieved using WoW-driven CCF (23.6%), while TCO yielded the lowest rate (4.5%). The combined TSP rate for interventions that adopted parallel W4SS + PDX screening was significantly higher than that of the W4SS-only screening interventions (22.6% vs. 7.0%; OR 3.9, 95% CI 3.74–3.98; *P* < 0.001). The NNS ranged from 46 for ACF nomads to 220 for TCO. The average NNT for interventions that used parallel W4SS + PDX screening was 4 (range: 4–5), while that of W4SS-only screening interventions was 14 (range: 11–22). Details on TSP, NNS, NNT, etc., are shown in Table 3.

**TABLE 3:** Comparison of TB case yield across the ACF interventions

	W4SS-only screening interventions					W4SS and PDX screening interventions				
	ICF public	ICF private	TCO	ACF nomads	CI	All W4SS-only interventions	PDX-driven ACF	WoW-driven CCF	All W4SS and PDX interventions	Total (all interventions)
Intervention categories										
Persons screened	12,632,167	6,003,098	2,197,142	23,903	552,966	21,409,276	127,994	167,399	295,393	21,704,669
Presumptive identified	824,079	602,337	238,871	8,597	137,466	1,811,350	12,357	10,740	23,097	1,834,447
Presumptive evaluated for TB	756,507	554,917	219,273	8,332	131,016	1,670,045	12,342	10,676	23,018	1,693,063
Cases diagnosed										
True screen +ve										
Total, *n* (%)	66,122 (8.7)	31,279 (5.6)	9,972 (4.5)	520 (6.2)	9,363 (7.1)	117,256 (7.0)	2,674 (21.7)	2,522 (23.6)	5,196 (22.6)	122,452 (7.2)
Female	26,763	12,308	4,280	179	4,128	47,658	782	634	1,416	49,074
Male	39,359	18,971	5,692	341	5,235	69,598	1,892	1,888	3,780	73,378
False screen positives, * n* (%)	690,385 (91.3)	523,638 (94.4)	209,301 (95.5)	7,812 (93.8)	121,653 (92.9)	1,552,789 (93.0)	9,668 (78.3)	8,154 (76.4)	17,822 (77.4)	1,570,611 (92.8)
NNS	191	192	220	46	59	183	48	66	57	177
NNT	11	18	22	16	14	14	5	4	4	14

ACF = active case-finding; ICF = intensified case-finding; TCO = targeted community outreaches; CI = contact investigation; W4SS = WHO 4-symptom screen; +ve = positive; PDX = portable digital X-ray; WoW = Wellness on Wheels; NNS = number needed to screen; NNT = number needed to test.

## DISCUSSION

This study compared the TB yield of the seven TB case-finding interventions conducted by KNCV Nigeria in 14 states of Nigeria. All interventions applied the principle of systematic identification of persons presumed to have TB in a predefined target population within and outside the health facility using the rapidly applied TB screening (W4SS with/or without AI-enabled PDX), followed by TB diagnostic testing and linkage to treatment, as appropriate.^16^ The study found that the interventions using a parallel W4SS + PDX TB screening algorithm were associated with a lower pre-diagnostic loss of those presumed to have TB and higher TB yield (i.e., higher TSP rate and lower NNT).

The study suggests that the ACF interventions employed by KNCV Nigeria for systematic TB screening in Nigeria had had a wide coverage, given that about 21.7 million persons, representing about 11% of the Nigerian population, were screened for TB within 27 months. Unfortunately, the project missed 8% of the presumptive TB identified before TB diagnostic evaluation could be conducted (Table 2), which may not be unusual in such a large project.^6^,^13^ Compared to a pre-diagnostic presumptive drop-out rate of 41% reported from a health centre in Uganda,^15^ this project’s performance in terms of pre-diagnostic drop-out rate (range: 3.1–8.2%) may be considered exceptional, considering the large population screened. Nevertheless, given the average TSP rate of 7.2% identified in the study, more effort should be made through repeated project evaluation to reduce the evaluation gap further and its impact on the spread of disease in the community.^7^ The community health implication of pre-diagnostic drop-out among those with presumptive TB is clearer when we note that 8 out of every hundred could be missed TB cases.

Furthermore, this study suggests that the odds of pre-diagnostic drop-out of persons with presumptive TB was about 25 times higher among ACF interventions that employ the W4SS-only screening algorithm than those that use a combination of W4SS and PDX screening. It is noteworthy that the 6-month WoW truck-driven CCF pilot project among hard-to-reach Niger Delta communities of Nigeria did not have any pre-diagnostic drop-out of presumptive TB.^5^ It is therefore likely that the exposure of clients to CXR during TB screening psychologically motivates identified presumptive patients to accept further TB evaluation, unlike the symptom-only-based screening that does not involve any form of investigation.

The TB case burden identified in this project is far higher than the national estimate of 219/100,000 population,^8^ which suggests that KNCV Nigeria ACF interventions are effective systematic screening programmes within the target communities. It has been shown that symptom screening, combined with CXR, improves the sensitivity and specificity of the TB screening algorithm;^11^ which may explain the increased TB case yield (i.e., TSP rate) among interventions that combine W4SS and PDX screening compared to symptom-only screening interventions (Table 3). Specifically, this study shows that presumptive TB identified from a W4SS + PDX screen-based intervention would yield about four times the number of TB cases than symptom-only-based screening interventions. This observed increase in TB case yield suggests that the W4SS + PDX screen-based interventions have a more efficient ACF process worthy of scale-up.

Although all the 14 states involved in the TB LON 1 and 2 region projects are high TB burden states, the target communities where each intervention was deployed were not homogenous; therefore, the NNS would not be a very reliable comparative indicator for the interventions. On the other hand, the NNT and the TSP rate target those with presumptive TB, a population selected after TB screening; it is therefore expected that interventions with a more accurate TB screening process would produce a higher TSP rate and lower NNT. This expectation is supported by previous reports, which showed that any symptom screening such as the W4SS, is sensitive, but its combination with CXR improves TB screening sensitivity and specificity.^11^,^17^ This study’s findings, therefore, suggest that the PDX-driven ACF and WoW-driven CCF have a more accurate TB screening process – both interventions had a combined average NNT of 4, which is 3.5 times lower than that of the interventions using the W4SS-only TB screening algorithm (Table 3). Likewise, the average TSP rate of 22.6% for the PDX-driven ACF and WoW-driven CCF is 3.2 times higher than that of the W4SS-only-based interventions.

This study was derived from the retrospective review of project data which restricted the outcomes assessed. For example, predictors of pre-diagnostic loss of persons with presumptive TB and characteristics of these persons, could not be assessed. However, it is strengthened by the fact that the assessment of the efficiency of key ACF interventions deployed by Nigeria’s NTP is a novel effort in Nigeria. The large data set used for programme assessment is also a strength. The study finding is thus expected to drive TB control policy in Nigeria and beyond.

In conclusion, ACF interventions in Nigeria utilising AI-enabled PDX machines in addition to W4SS for TB screening had the least pre-diagnostic loss of presumptive persons and higher yield of TB cases when compared with interventions using W4SS-only TB screening. Thus, PDX-driven ACF and WoW-driven CCF were the most efficient of all the seven ACF interventions in use by KNCV Nigeria for the LON region 1 & 2 project. We recommend the incorporation of AI-enabled PDX into every ACF intervention in Nigeria to improve TB case yield.
